# Supplementary material for: Content-rich biological network constructed by mining PubMed abstracts
Source: BMC Bioinformatics. 2004 Oct 8;5:147. doi: 10.1186/1471-2105-5-147 (PMC528731; doi:10.1186/1471-2105-5-147)
Supplement: Additional File 2 — The original results of the above study (non-essential files are deleted to keep the file size under the limit set by BMC bioinformatics). [file 1471-2105-5-147-S2.bz2 › chilibotAdditionalFile2/dip20/530ID9671457E10847/html/PGR_HMGB1.html]

 


 **PGR** and **HMGB1** 
  
Found 11 abstracts in PubMed, retrieved 11.  
 

 What does Google say? 
 PDF only 
| .edu only 

---

**Interactive relationship** (e.g. stimulation, inhibition, etc)

**Stimulatory relationship**- The DNA bending protein HMG 1  [ **HMGB1** ]  enhances progesterone receptor  [ **PGR** ]  binding to its target DNA sequences.  Ref: 8164686 Mol Cell Biol, 1994
**Neutral relationship**- We have previously reported that progesterone receptor  [ **PGR** ]  requires a nuclear factor, high mobility group 1 or 2 HMG 1  [ **HMGB1** ]  2 for high affinity interaction with DNA in vitro and for full transcriptional activity in vivo.  Ref: 10503712 Steroids, 1999

**Non-interactive relationship** (e.g. studied together, co-existance, homology, etc.)

- After gonadotrophin releasing hormone analogue HMG  [ **HMGB1** ]  superovulation, the progesterone receptor  [ **PGR** ]  PR has been found less frequently in progesterone supplemented cycles than in non supplemented cycles.  Ref: 8745303 Hum Reprod, 1995
- This DNA bending activity appears to be central to at least some of the recently elucidated functions of HMG 1  [ **HMGB1** ]  2, such as the enhancement of progesterone receptor  [ **PGR** ]  DNA binding.  Ref: 7706284 J Biol Chem, 1995
- We previously reported that the chromatin high mobility group protein 1  [ **HMGB1** ]  HMG 1  [ **HMGB1** ]  enhances the sequence specific DNA binding activity of progesterone receptor  [ **PGR** ]  PR in vitro, thus providing the first evidence that HMG 1  [ **HMGB1** ]  may have a coregulatory role in steroid receptor mediated gene transcription.  Ref: 9671457 Mol Cell Biol, 1998
